# Emerging Sedation Strategies in Pediatric Emergency Care: Pharmacological Advances and Clinical Applications

**DOI:** 10.7759/cureus.105779

**Published:** 2026-03-24

**Authors:** Sabrina Montoya, Diego Alvarez Ramirez, Ronald Chavarría, Elia L Zamora, Christian Andrés Soto Cordero

**Affiliations:** 1 Emergency Department, Hospital Monseñor Sanabria Martínez, Puntarenas, CRI

**Keywords:** airway safety, dexmedetomidine, emergency medicine, pediatric procedural sedation, remimazolam, ultra-short-acting agents

## Abstract

Emerging sedative agents are increasingly being explored to improve the safety and effectiveness of procedural sedation in pediatric emergency care. Traditional agents such as propofol and ketamine remain widely used because of their rapid onset and clinical effectiveness; however, they may be associated with adverse effects, including hypotension, respiratory depression, and emergence reactions, which require careful monitoring during pediatric procedural sedation. In this context, newer pharmacologic approaches have been investigated to optimize safety and procedural control. Remimazolam, a benzodiazepine metabolized by tissue esterases, demonstrates rapid clearance, minimal drug accumulation, and stable hemodynamic profiles. Clinical studies suggest comparable efficacy to propofol in procedural sedation, with potential advantages including reduced respiratory depression and a lower incidence of emergence delirium. Its reversibility with flumazenil may further enhance safety in selected clinical scenarios. Dexmedetomidine, a selective α₂-adrenergic receptor agonist, provides sedation that resembles natural sleep and offers intrinsic analgesic properties with minimal respiratory depression. Although its onset of action may be slower than that of agents such as propofol, dexmedetomidine is associated with favorable respiratory stability and may be particularly useful in pediatric patients requiring cooperative or prolonged sedation. Intranasal administration represents a noninvasive alternative in situations where intravenous access has not yet been established, although its pharmacokinetic profile may result in delayed onset. These agents have been applied in a variety of pediatric procedures, including painful interventions, imaging studies, and short diagnostic procedures. Their use may be particularly valuable in children with developmental disorders or complex comorbidities, where stable hemodynamic and respiratory profiles are essential. Contemporary safety frameworks emphasize structured risk assessment, airway preparedness, weight-based dosing strategies, and continuous monitoring techniques such as capnography and pulse oximetry. Current evidence from randomized and observational studies suggests that emerging sedative strategies may provide effective procedural sedation while maintaining favorable safety profiles when appropriately integrated into multimodal sedation approaches. Nevertheless, important knowledge gaps remain, including limited pediatric data for certain agents, the need for standardized protocols, and the evaluation of long-term outcomes in younger patient populations.

## Introduction and background

Procedural sedation plays a central role in pediatric emergency care by allowing children to undergo diagnostic and therapeutic interventions safely while minimizing distress and procedural discomfort. Adequate sedation facilitates immobilization, reduces anxiety, and provides effective analgesia, all of which are essential for completing procedures efficiently and with minimal psychological or physical trauma [1]. Several pharmacologic agents are currently used in pediatric procedural sedation, including propofol, ketamine, benzodiazepines, and α₂-adrenergic receptor agonists. Among these, propofol and ketamine remain widely employed because of their rapid onset and high procedural success rates. However, their use may be associated with adverse effects such as hypotension, respiratory depression, or emergence reactions, which require careful monitoring and appropriate airway preparedness in emergency settings [2,3].

In recent years, increasing attention has been directed toward newer sedative agents with pharmacokinetic profiles that may allow greater procedural control and faster recovery following sedation. Remimazolam, a benzodiazepine metabolized by tissue esterases, has been proposed as a promising alternative due to its rapid clearance, minimal drug accumulation, and metabolism largely independent of hepatic function [1]. These characteristics may permit more predictable titration and recovery compared with traditional benzodiazepines. Additionally, its reversibility with flumazenil provides an additional safety consideration in selected clinical scenarios [4]. Early clinical investigations have suggested that remimazolam may achieve sedation efficacy comparable to propofol in certain procedural contexts while maintaining relatively stable hemodynamic and respiratory profiles [5].

Dexmedetomidine represents another pharmacologic option that has gained increasing use in pediatric sedation. As a selective α₂-adrenergic receptor agonist, dexmedetomidine produces sedation resembling natural sleep while preserving spontaneous ventilation and providing intrinsic analgesic properties [1]. Although its onset of action is generally slower than that of agents such as propofol, its favorable respiratory stability may be advantageous in selected pediatric patients. Intranasal administration has also been explored as a noninvasive alternative in situations where intravenous access has not yet been established, particularly in younger children [6]. In addition to single-agent sedation, combination strategies involving agents such as propofol and S(+)-ketamine have been investigated in order to balance sedative depth, analgesia, and recovery characteristics, illustrating the evolving landscape of pharmacologic approaches in pediatric procedural sedation [7]. The aim of this review is to analyze emerging pharmacologic strategies for procedural sedation in pediatric emergency care, with particular attention to agents with favorable pharmacokinetic characteristics. The discussion focuses on their safety profiles, pharmacological mechanisms, and clinical applications, as well as their potential role in optimizing sedation practices and reducing adverse events in acute pediatric settings.

## Review

Methods

This study was conducted as a structured narrative review aimed at synthesizing contemporary evidence on emerging pharmacologic strategies for procedural sedation in pediatric emergency care. The review was designed to integrate pharmacological mechanisms, clinical effectiveness, safety profiles, and practical applicability within emergency settings. Rather than employing systematic meta-analytic techniques, the methodological approach prioritized interpretative synthesis and clinical relevance, with particular focus on pharmacologic agents that may offer improved procedural control and recovery characteristics in pediatric procedural sedation.

A literature search was performed in PubMed, Scopus, and Web of Science, selected for their extensive coverage in anesthesiology, emergency medicine, and pediatric research. Peer-reviewed articles published between January 2020 and December 2025 in English or Spanish were considered. This timeframe was selected to capture recent developments in pediatric sedation practices, including the clinical evaluation of newer agents such as remimazolam and evolving safety standards in emergency procedural sedation.

The search strategy combined Medical Subject Headings and free-text terms using Boolean operators. Representative search terms included combinations of (“pediatric procedural sedation” OR “pediatric emergency sedation”), (“remimazolam” OR “dexmedetomidine” OR “procedural sedation agents”), and (“airway safety” OR “adverse events” OR “monitoring”) within the context of emergency medicine. The initial search yielded 164 records. After removal of duplicates and screening of titles and abstracts for relevance, 72 articles were selected for full-text evaluation, of which 36 met the eligibility criteria and were included in the final qualitative synthesis. Study selection was performed by two authors, and discrepancies were resolved through discussion and consensus.

Eligible sources included randomized controlled trials, multicenter observational studies, systematic reviews, meta-analyses, pharmacokinetic and pharmacodynamic investigations with clinical applicability, and consensus guidelines from recognized scientific societies. Studies limited exclusively to adult populations, non-translational experimental models, duplicate publications, or investigations lacking clinically relevant safety or outcome data were excluded.

Given the narrative design of the review, methodological quality was assessed qualitatively rather than through formal risk-of-bias scoring tools. Particular attention was given to study design, sample size, reported safety outcomes, definitions of adverse events, and applicability to pediatric procedural sedation. Heterogeneity among studies, including differences in age groups, procedural contexts, monitoring standards, and sedation protocols, was considered during the interpretative synthesis. When conflicting findings were identified, greater interpretative weight was given to higher-level evidence and guideline-supported recommendations.

As a narrative review, this study does not provide pooled quantitative estimates and may be subject to selection bias inherent to narrative synthesis methodologies. Artificial intelligence tools were used solely to assist with structural organization and language refinement. Scientific evaluation of the literature and final interpretation of the evidence were performed independently by the authors to maintain academic rigor.

Pharmacological basis of ultra-short-acting agents

The clinical utility of several emerging sedative agents in pediatric procedural sedation is largely determined by pharmacologic mechanisms that allow rapid onset of action, controllable depth of sedation, and relatively predictable recovery profiles. Remimazolam, a benzodiazepine that acts through modulation of γ-aminobutyric acid type A receptors, produces sedation, anxiolysis, and amnesia similar to other agents in its class. However, its pharmacologic behavior differs from traditional benzodiazepines because it undergoes metabolism by tissue esterases, a process that is largely independent of hepatic or renal function. This metabolic pathway contributes to a short context-sensitive half-life of approximately 10 minutes, which may facilitate titration and recovery in dynamic clinical environments [1,8].

Dexmedetomidine represents another pharmacologic option increasingly used in pediatric sedation. As a selective α₂-adrenergic receptor agonist, it produces a sedative state resembling natural sleep while preserving spontaneous ventilation and providing intrinsic co-analgesic effects. Its characteristic “arousable sedation” profile allows patients to remain responsive to stimulation, which may be advantageous in emergency settings where continuous neurological or clinical assessment is required [1]. Unlike agents with very rapid onset such as propofol, dexmedetomidine typically demonstrates a slower onset of sedation but maintains favorable respiratory stability.

Pharmacokinetic investigations in pediatric populations provide additional insight into the behavior of these agents during procedural sedation. Remimazolam demonstrates relatively high clearance and a small steady-state volume of distribution in children, characteristics that may support rapid achievement of therapeutic levels and relatively prompt recovery. Pharmacokinetic modeling studies suggest that a three-compartment model best describes its disposition, with age and body weight serving as important determinants of drug distribution and clearance [9,10]. Although the available pediatric evidence remains limited, early clinical studies have reported generally stable hemodynamic and respiratory profiles during procedural use [2,3].

Dexmedetomidine pharmacokinetics have also been evaluated in pediatric populations. Intranasal administration can achieve clinically relevant plasma concentrations in young children, and its disposition has been described using a two-compartment pharmacokinetic model. This noninvasive route of administration has contributed to its increasing use in situations where intravenous access has not yet been established or where less invasive sedation strategies are desirable [11].

When compared with conventional sedatives such as propofol or ketamine, these agents offer distinct pharmacologic characteristics that may influence their clinical applications. Remimazolam may provide a relatively predictable recovery profile due to its esterase-mediated metabolism, whereas dexmedetomidine offers sedation with preserved respiratory function. However, the choice of agent remains dependent on procedural requirements, patient characteristics, and clinician experience, and further studies are needed to clarify their optimal role in pediatric procedural sedation [7,12].

Current ultra-short-acting agents in pediatric emergency practice

Remimazolam has recently gained attention as a potential option for procedural sedation in pediatric emergency settings. As a benzodiazepine metabolized by tissue esterases, it demonstrates rapid onset and relatively predictable recovery characteristics compared with traditional benzodiazepines. Its metabolism, which is largely independent of hepatic function, may allow more consistent pharmacokinetic behavior across different patient populations. Additionally, the ability to reverse its sedative effects with flumazenil provides an additional safety consideration in situations where rapid recovery of consciousness may be required [2,13]. Early clinical investigations suggest that remimazolam can achieve effective sedation with generally stable hemodynamic and respiratory profiles, although pediatric-specific evidence remains limited and further studies are needed to better define its role in emergency procedural sedation [5,13,14].

Remifentanil, a short-acting opioid with rapid metabolism through nonspecific plasma esterases, is frequently incorporated into multimodal sedation strategies because of its potent analgesic properties and brief duration of action. Although dedicated pediatric procedural sedation studies remain relatively limited, remifentanil is often used as an adjunct to sedative agents such as propofol or benzodiazepines in order to enhance analgesia during short painful procedures. Its short context-sensitive half-life allows tight titration and rapid offset, characteristics that may be advantageous in emergency settings where rapid procedural completion and recovery are desired [15].

Propofol continues to play a central role in pediatric emergency sedation due to its rapid onset and reliable sedative effect. In many clinical settings, clinicians employ titrated dosing strategies, sometimes described as micro-dosing protocols, in which small incremental boluses are administered to achieve the desired depth of sedation while minimizing the risk of hypotension or respiratory depression. These approaches allow clinicians to maintain the advantages of propofol while improving hemodynamic stability during procedural sedation. In certain cases, propofol may also be combined with other agents, such as low-dose ketamine or benzodiazepines, to balance analgesia, sedation depth, and recovery characteristics [16].

Combination sedation strategies have also been explored to optimize procedural conditions while reducing dose-dependent adverse effects. One commonly described approach is the ketamine-propofol combination, often referred to as ketofol. By administering lower doses of each agent, this strategy aims to balance the sedative and analgesic properties of ketamine with the rapid onset and recovery characteristics of propofol. Such multimodal approaches may help reduce the incidence of adverse effects associated with higher single-agent dosing, including ketamine-related emergence reactions or propofol-associated cardiorespiratory depression. Although ketofol has been studied extensively in adult populations, pediatric evidence continues to evolve, and careful patient selection and monitoring remain essential in emergency care settings [2].

Clinical applications and indications

Sedative and analgesic strategies play a central role in facilitating painful procedures in pediatric emergency settings. For interventions such as laceration repair and fracture reduction, agents including ketamine and opioids such as fentanyl remain widely used because of their rapid onset and reliable analgesic properties. Intranasal ketamine has demonstrated utility in selected procedural settings, achieving effective sedation in a substantial proportion of laceration-repair cases with a relatively low incidence of adverse events [17,18]. Similarly, esketamine has been reported as effective during forearm fracture reductions, demonstrating high procedural success rates in small clinical series, although broader pediatric evidence remains limited [19].

Imaging procedures such as magnetic resonance imaging and computed tomography require sedation strategies that maintain immobility while preserving respiratory stability. Dexmedetomidine has been increasingly used in these contexts because it can provide relatively stable sedation with minimal respiratory depression. Evidence from clinical sedation programs has demonstrated high procedural success rates during magnetic resonance imaging examinations when dexmedetomidine-based protocols are used [20]. Intranasal administration may represent a useful alternative in children who do not yet have intravenous access, potentially reducing procedural distress and facilitating sedation initiation in selected cases [21].

For brief diagnostic interventions, remimazolam has been explored as a potential sedative option due to its rapid metabolism by tissue esterases and relatively short duration of action. These pharmacokinetic characteristics may support rapid titration and recovery during short procedures. Early clinical evaluations suggest that remimazolam can achieve effective sedation with generally stable hemodynamic profiles, although adjunctive analgesic or sedative agents such as ketamine may still be required depending on procedural requirements [1,2,12].

Special considerations are necessary in children with developmental disorders, chronic illnesses, or complex medical conditions. Dexmedetomidine has been reported to be well tolerated in children with neurodevelopmental disorders, producing a sedation state resembling natural sleep and potentially reducing agitation during procedures [17]. Remimazolam’s rapid clearance and limited drug accumulation may also make it a useful option in patients with altered organ function; however, pediatric-specific evidence in medically complex populations remains limited, and further research is required to better define its safety and optimal use in these groups [1,15].

Safety frameworks and monitoring standards

Effective and safe pediatric procedural sedation requires a comprehensive pre-sedation assessment to identify patients who may be at increased risk for complications. This evaluation typically includes factors such as age, body weight, comorbid conditions, prior anesthetic exposure, airway anatomy, and the characteristics of the planned procedure. A structured risk stratification approach helps clinicians determine the most appropriate sedation strategy and tailor drug selection and dosing to the needs of each patient, thereby improving procedural success and safety [1,21]. Emerging sedative agents with relatively predictable pharmacokinetic profiles, including remimazolam and dexmedetomidine, have attracted attention because they may allow more controlled titration and recovery in selected procedural contexts [1,22].

Airway management preparedness remains a fundamental component of pediatric sedation practice. Even when agents with favorable respiratory profiles are used, clinicians must remain prepared to manage airway complications. For example, remimazolam’s relatively short duration of action and reversibility with flumazenil may facilitate recovery in situations where rapid restoration of consciousness is desirable. Nevertheless, standard safety protocols require the presence of trained personnel capable of performing pediatric airway maneuvers, as well as immediate availability of equipment such as suction devices, bag-valve masks, and advanced airway adjuncts to ensure prompt intervention should respiratory compromise occur [1,2].

Appropriate dosing strategies are essential to achieve effective sedation while minimizing adverse effects. Dexmedetomidine dosing in pediatric patients is typically adjusted according to age and body weight, as pharmacokinetic differences between infants, children, and adolescents may influence drug exposure [22]. Remimazolam dosing protocols in children are still evolving and are often adapted from adult dosing strategies with weight-based modifications, although further pediatric-specific studies are needed to better define optimal dosing regimens [2].

Continuous physiologic monitoring is a critical component of safe pediatric sedation. Standard monitoring typically includes pulse oximetry, noninvasive blood pressure measurement, and electrocardiographic monitoring. Capnography is increasingly recommended because it allows early detection of hypoventilation or apnea before oxygen desaturation occurs. In selected situations, supplemental oxygen delivery systems such as high-flow nasal oxygen may also be used to support oxygenation during sedation, particularly in patients at increased risk for respiratory compromise [1,2].

Adverse events and risk mitigation

Respiratory safety is a central consideration in pediatric procedural sedation. Certain sedative agents, including remimazolam and dexmedetomidine, have attracted attention because they may be associated with relatively low rates of respiratory depression compared with some traditional sedatives when used in appropriately monitored settings. Dexmedetomidine, in particular, is known for producing sedation while generally preserving spontaneous ventilation, which may be advantageous in children with underlying respiratory vulnerabilities or in procedures where maintenance of airway reflexes is desirable [1]. In addition, multimodal sedation strategies may help optimize respiratory safety. For example, adjunctive use of agents such as esketamine has been explored in combination protocols and may contribute to improved analgesia while potentially reducing the need for higher doses of single sedative agents [23].

**Figure 1:**
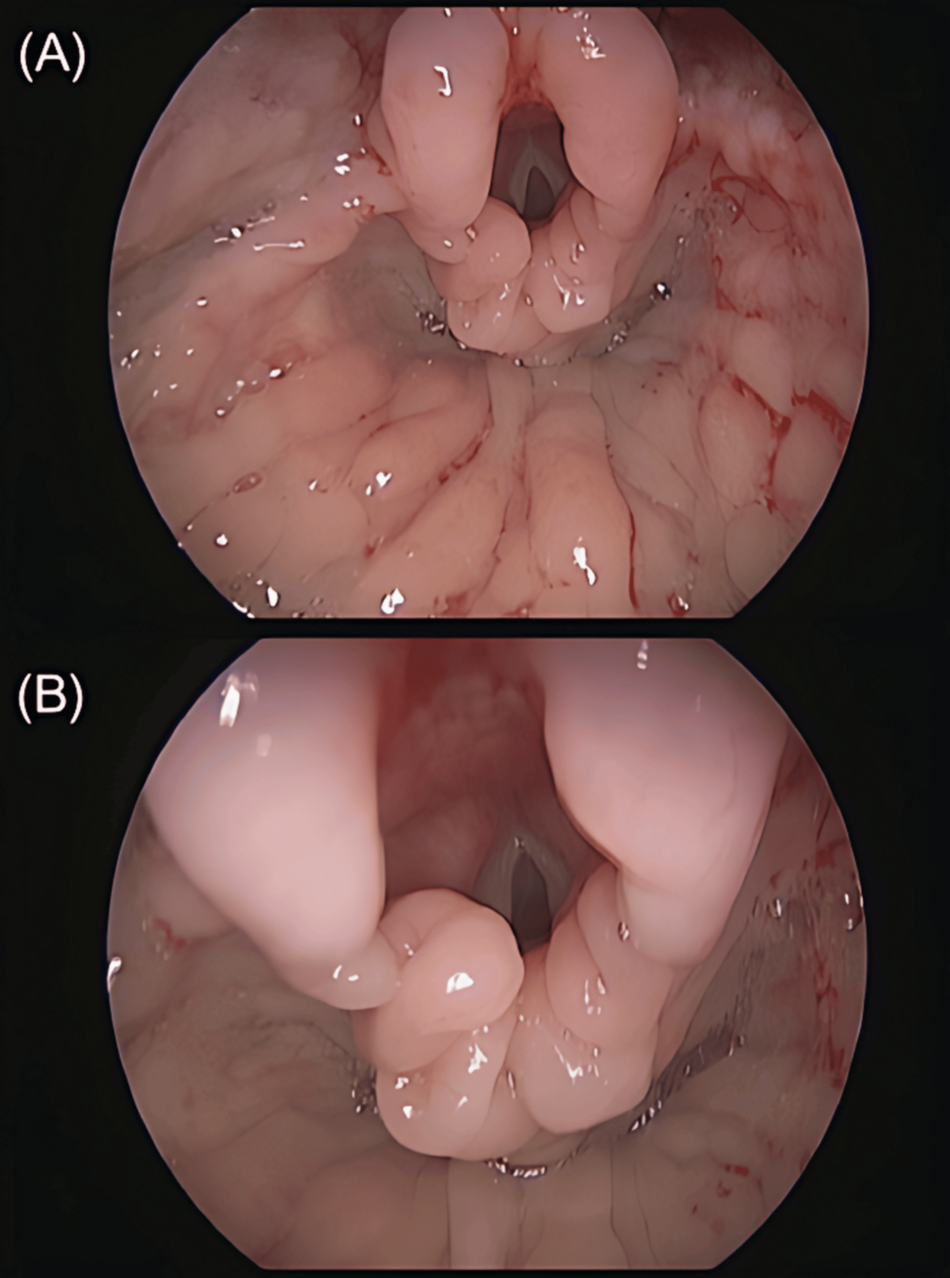
Endoscopic visualization of supraglottic improvement following airway stabilization. A. Improved supraglottic swelling with no inspiratory prolapse into the endoglottis. B. Zoomed view of the arytenoid region showing reduction in the size of previously prominent tissues. Figure reproduced from Rajasekaran et al. (Creative Commons Attribution-NonCommercial-NoDerivs (CC BY-ND)) [24].

The respiratory considerations associated with pediatric sedation are illustrated in Figure 1, which demonstrates endoscopic visualization of supraglottic airway anatomy following stabilization measures. The image highlights improved supraglottic patency with reduced inspiratory prolapse and decreased tissue prominence, features that are relevant to airway management during procedural sedation.

Hemodynamic stability is another important consideration during pediatric sedation. Remimazolam has been associated in several clinical studies with relatively stable cardiovascular profiles during procedural sedation, although direct comparisons with other sedatives should be interpreted cautiously due to differences in study design and patient populations [16]. Some pediatric studies have reported modest variations in mean arterial pressure during sedation; however, these changes have generally remained within clinically manageable ranges and rarely required pharmacologic intervention [2]. Dexmedetomidine may also provide relatively stable hemodynamic conditions in many patients, although clinicians should remain aware that bradycardia and hypotension can occur, particularly with higher doses or rapid administration [1].

Emergence phenomena, including emergence delirium, are recognized complications in pediatric anesthesia and sedation. Some studies have suggested that remimazolam-based sedation protocols may be associated with lower rates of emergence delirium compared with certain traditional sedatives, although the available pediatric data remain limited and further comparative studies are needed [16,25]. Dexmedetomidine has also been used in both prevention and management of agitation or delirium during the recovery phase due to its sedative and sympatholytic properties [1].

Several pharmacologic characteristics may facilitate rapid recovery following sedation. Remimazolam undergoes rapid metabolism by tissue esterases, which may allow relatively prompt return to baseline consciousness compared with longer-acting benzodiazepines. Additionally, its effects can be reversed with flumazenil when clinically indicated [1]. Adjunctive use of low-dose opioids such as alfentanil has been explored in certain procedural settings to reduce the amount of sedative required to achieve adequate conditions, although careful titration and monitoring remain essential to minimize adverse events [26]. These strategies may support efficient recovery and patient turnover in high-volume pediatric emergency environments while maintaining appropriate safety standards [2].

Integration of multimodal sedation approaches

The integration of pharmacologic sedation strategies with supportive non-pharmacologic measures represents an evolving approach in pediatric emergency sedation. These multimodal strategies aim to optimize procedural conditions while minimizing exposure to higher doses of single sedative agents. Remimazolam, a benzodiazepine metabolized rapidly by tissue esterases, has attracted interest for short procedures because of its relatively rapid clearance and predictable recovery characteristics. In some clinical settings, remimazolam may be used in combination with agents such as propofol or ketamine to achieve deeper sedation or improve procedural conditions when analgesia is required. However, the choice of combination therapy should be individualized according to procedural needs, patient characteristics, and clinician experience [1,2].

Non-pharmacologic interventions also play an important role in pediatric sedation strategies. Techniques such as distraction, caregiver presence, and behavioral support can reduce anxiety and improve procedural cooperation, potentially decreasing the required doses of sedative medications. These approaches are particularly valuable in children undergoing minimally invasive procedures or imaging studies, where maintaining calm cooperation can significantly improve procedural success [27].

Minimal sedation and anxiolysis protocols may incorporate agents with favorable respiratory profiles. Dexmedetomidine is often used in these contexts because it produces a sedative state resembling natural sleep while generally preserving spontaneous ventilation. Intranasal dexmedetomidine has been explored as a noninvasive option in children who do not yet have intravenous access, allowing sedation to begin without immediate vascular instrumentation in selected situations [1,21]. Additional adjunctive measures, including oral melatonin, have been investigated for their anxiolytic and calming effects, with some studies suggesting potential reductions in peri-procedural agitation and improved pre-procedural comfort [28]. Similarly, sub-anesthetic doses of esketamine may be used as an adjunct to provide analgesia and reduce the need for higher doses of primary sedatives during certain procedures [23].

In resource-limited emergency departments, pragmatic and adaptable sedation strategies are particularly important. Intranasal administration of dexmedetomidine or ketamine has been explored as an alternative when intravenous access is difficult or delayed. Combination approaches such as dexmedetomidine with ketamine (sometimes referred to as ketodex) have been described in small clinical studies and may provide adequate sedation and analgesia in selected procedural contexts [6]. In certain situations where standard sedative agents are unavailable or contraindicated, enteral pentobarbital has also been used as an alternative option for sedation in critically ill pediatric patients, although its use requires careful monitoring [29]. Combination strategies such as dexmedetomidine with propofol may allow dose reduction of individual agents in some procedural settings, but careful titration and monitoring remain essential to minimize adverse effects [30].

Evidence from recent clinical trials and guidelines

Recent randomized and observational studies have expanded the evidence base evaluating emerging sedative agents in pediatric procedural sedation. Remimazolam has attracted increasing attention because of its rapid metabolism by tissue esterases, limited drug accumulation, and relatively predictable recovery profile. Early clinical studies suggest that remimazolam can achieve effective sedation with generally stable hemodynamic and respiratory profiles during short procedures. Some investigations have also reported lower rates of emergence delirium when compared with certain traditional sedatives; however, these findings should be interpreted cautiously given the limited number of pediatric trials and differences in study design [1,7].

Dexmedetomidine has likewise been investigated in multiple pediatric sedation settings. Comparative studies with midazolam have demonstrated reduced emergence agitation and improved sedation quality during parental separation in some pre-procedural contexts. Nevertheless, evidence regarding its advantages in other clinical scenarios, such as mask induction or postoperative analgesia, remains variable across studies [31]. Intranasal dexmedetomidine has also been explored as a noninvasive sedation strategy in young children and has demonstrated acceptable sedation success rates in several clinical settings [32]. In addition, esketamine has been evaluated in pediatric anesthesia and sedation studies, with some investigations suggesting that doses around 0.3 mg/kg may reduce emergence agitation following procedures such as tonsillectomy and adenoidectomy, although further research is needed to confirm optimal dosing strategies [33].

Professional societies and pediatric formularies increasingly acknowledge the role of these agents within evolving sedation practices. Dexmedetomidine, although still considered off-label for several pediatric indications, is widely used in clinical practice for premedication and procedural sedation. Evidence-based dosing recommendations from sources such as the Dutch Pediatric Formulary highlight its sedative properties and practical clinical applications [22]. Broader pediatric and emergency medicine guidelines emphasize the importance of individualized dosing, continuous physiologic monitoring, and careful patient selection, particularly in high-risk populations such as infants or children with significant comorbidities [34].

Despite these developments, important knowledge gaps remain. Remimazolam currently lacks robust clinical data in infants younger than one year of age, and information regarding its potential long-term neurodevelopmental effects is limited [16]. Dexmedetomidine has demonstrated benefits in reducing agitation in several clinical contexts; however, further research is needed to clarify its effectiveness across diverse procedural scenarios and to determine whether repeated or prolonged exposure influences long-term outcomes [35]. Additionally, more direct comparative trials between emerging sedative agents and conventional sedatives would help clarify their relative advantages and support the development of standardized pediatric sedation protocols [36].

## Conclusions

Emerging sedative agents such as remimazolam and dexmedetomidine offer pharmacologic characteristics that may facilitate procedural sedation in pediatric emergency care. Their relatively predictable pharmacokinetic profiles, favorable respiratory stability, and generally manageable hemodynamic effects make them attractive options in selected procedural contexts, particularly when rapid titration and recovery are desirable. These agents have been increasingly explored across various clinical scenarios, including painful interventions, imaging procedures, and brief diagnostic evaluations. In many cases, their effectiveness may be enhanced when incorporated into multimodal sedation strategies that combine pharmacologic and non-pharmacologic approaches, allowing clinicians to tailor sedation plans according to individual patient needs and procedural requirements.

Current evidence from randomized trials, observational studies, and emerging clinical guidelines suggests that these agents may play an expanding role in pediatric procedural sedation. However, important knowledge gaps remain. Additional research is needed to better define optimal dosing strategies, evaluate their safety in vulnerable populations such as infants and medically complex children, and clarify their comparative effectiveness relative to established sedatives. Addressing these questions will help inform the development of more standardized and evidence-based sedation protocols in pediatric emergency settings.
